# Three Cottage Hospitals

**Published:** 1893-05-13

**Authors:** 


### THE INSTITUTIONAL WORKSHOP.

### WITHIN THE HOSPITALS.

### THREE COTTAGE HOSPITALS.

Cottage hospitals are again to the fore, and a third edition of the standard work on the subject is now in the press. We have thought it might be of interest, therefore, to visit a few of these institutions and briefly give the impression their present condition conveys to the mind of the trained visitor. The three hospitals we propose to deal with are those of Watford, Harrow-on-the-Hill, and Chesham. They are nearly all of a size, as not one of them contains more than ten beds. That at Watford may be considered mainly urban, and the other two mainly rural, so far as the character of the patients is concerned.

WATFORD COTTAGE HOSPITAL.

In a subsequent issue we propose to give a plan of this institution, because the architect has succeeded in so arranging the buildings as to make them as little costly and as convenient as it is possible to make a small institution specially built for cottage hospital purposes. The elevation is most attractive, the situation good, and the site, though relativley small, sufficient for the purpose. The institution, which contains nine beds, is worked by a Nurse-Matron, with one probationer and a servant. The arrangements are simple but adequate, the sanitary conditions are good, and if there were, as at Harrow, a Ladies' Committee who took a real personal interest in the management, we believe that the comfort of the patients and the efficiency of the institution would be materially benefited.

CHESHAM COTTAGE HOSPITAL.

This institution is situated on the hill above the town of Chesham. No more picturesque or healthful site can be imagined, and it is a grievous fact that the townspeople should have permitted the air of the institution to be befouled by the erection of some small gasworks, which emit smells of so bad a character as to convince us that the manufacture of gas under improved conditions is, to say the least, but little comprehended by those in charge. The hospital contains seven beds. It is built, as we have said, on the side of the cliff, and has been so planned as to make the frontage attractive. The architect's name did not appear, but these buildings eloquently testify to the importance of selecting one who has studied the subject of hospital construction. Everything seems to have been sacrificed to appearance, and in consequence the buildings are cramped and inconvenient, and spread over much too large a surface. The convalescent room is built at the end of a long passage, with three outside walls, and is practically never occupied, we believe, in consequence. The Committee use it occasionally, but otherwise it is useless. The wards are too narrow, and the floor space in consequence is cramped and inadequate. The patients, however, seem to do well. The Nurse-Matron is much interested in her work, and seems to do her best to make the whole place homely and comfortable. Here, as at Watford, although we understand there is a Ladies' Committee, we noticed evidence of a want of intelligent supervision in the domestic arrangements. We would suggest that the ladies of the Committee should visit the Harrow Cottage Hospital, ascertain what the rules are, and study its management. Then, we believe, the linen room and offices especially, will be placed in better order than they are at present.

HARROW-ON-THE-HILL COTTAGE HOSPITAL.

This institution is given high praise in the second edition of "Cottage Hospitals." That edition was published thirteen years ago, but the "Medical Directory" still contains no notice of the existence of the institution. We paid it a visit with genuine interest, and were delighted with its condition and efficiency. It is splendidly situated, has most attractive inviting gardens, and interiorly presents an atmosphere of comfort which must prove very satisfactory to the patients. It is under the management of a Ladies' Committee, consisting of seven ladies, and there is also a lady manager, Miss C. Hewlett, who has a room in the hospital, and visits it twice each day. The hospital is beautifully kept, and although the servant seems to have more than enough to do, in every other respect this little hospital, with its nine beds, leaves little, if anything, to be desired. The scheme of the hospital has been well arranged, and embraces a provident dispensary, with an out-patient room, and contains a convalescent room, a ward for patients who can pay a guinea a week, and is altogether well planned, well founded, and well kept. The provident dispensary not only gives each of the two doctors £100 a year, but provides them in part with an assistant, seeing that these gentlemen act as dispensers in turn, and that the institution pays £40 a year for dispensing. This provident dispensary, as well as the Harrow Cottage Hospital would appear to be a model which medical men in rural districts might wisely follow all over the country, to their own advantage and to the advantage of the poor people. It is interesting to note that, whereas the old payment used to be 2s. 6d. per week for a patient during residence in the Cottage Hospital, the three institutions we have described regard 2s. 6d. as a minimum payment, and very frequently receive 5s. a week per patient. How long will the general hospitals continue the plan of indefinitely enlarging the in-patient department, and of receiving an ever-increasing number of persons as free in-patients, a large proportion of whom are far better able to pay partially or wholly for the treatment they receive than the much more humble and poorer persons who frequent the wards of the Cottage Hospital?

